# Curtailing the Path From Epistaxis to Genetics: The Diagnostic Value of Detailed Medical History in Hereditary Macrothrombocytopenia

**DOI:** 10.7759/cureus.109725

**Published:** 2026-05-27

**Authors:** Lucija Ruzman, Emilia Köpcke, Izabela Kranjcec

**Affiliations:** 1 Department of Pediatrics, Division of Hematology and Oncology, Clinical Hospital Centre Rijeka, Rijeka, HRV; 2 Department of Pediatrics, Faculty of Medicine, University of Rijeka, Rijeka, HRV; 3 Department of Oncology and Hematology, Children's Hospital Zagreb, Zagreb, HRV; 4 Department of Pediatrics, University North, Varaždin, HRV

**Keywords:** epistaxis, exome sequencing, genetic testing, hereditary, thrombocytopenia

## Abstract

Hereditary macrothrombocytopenias represent a heterogeneous group of inherited platelet disorders that may present with variable, often mild, bleeding symptoms and are frequently misdiagnosed as acquired thrombocytopenias. This report presents a four-year-old boy with recurrent severe epistaxis and a family history of increased bleeding tendencies accompanied by low platelet counts. The patient had mild thrombocytopenia, large platelets, and a slightly prolonged platelet function analysis, while standard coagulation studies and thromboelastography were within normal limits. Next-generation sequencing identified a heterozygous variant in the alpha-actinin-1 (ACTN1) gene, confirming the diagnosis of ACTN1-related thrombocytopenia. Management consisted of supportive care and observation. This case underscores the significance of genetic testing in the diagnostic evaluation of suspected hereditary thrombocytopenias to facilitate accurate diagnosis, prevent unnecessary interventions, and enable appropriate genetic counseling for affected families.

## Introduction

Hereditary macrothrombocytopenias are a group of rare genetic disorders characterized by thrombocytopenia with enlarged platelets and variable bleeding symptoms, including disorders such as Bernard-Soulier syndrome (BSS), MYH9-related disease, and gray platelet syndrome [1]. They are frequently misdiagnosed as immune thrombocytopenia (ITP) or other acquired conditions [2]. One autosomal dominant form is alpha-actinin-1 (ACTN1)-related thrombocytopenia (ACTN1-RT; Online Mendelian Inheritance in Man: 615193), caused by pathogenic variants in the ACTN1 gene. This condition typically presents with absent or mild bleeding tendency, reduced platelet counts with large platelets, and platelet size variation without significant in vitro functional abnormalities [3].

We report the case of a boy with recurrent spontaneous epistaxis since infancy, a strong maternal history of bleeding tendencies, and mildly decreased, intermittently normal platelet counts. After exclusion of secondary causes of thrombocytopenia, next-generation sequencing (NGS) identified a likely pathogenic variant in the ACTN1 gene, confirming the diagnosis of hereditary macrothrombocytopenia.

## Case presentation

A four-year-old boy presented to the outpatient clinic for evaluation of frequent and severe epistaxis. A detailed medical history revealed that since the first year of life, he had experienced daily nosebleeds lasting up to 20 minutes, often soaking tissues and requiring prolonged compression to achieve hemostasis, which later decreased in frequency to several episodes every few months. The episodes occurred spontaneously, often during the night. His parents also reported occasional hematomas disproportionate to the degree of trauma in both size and distribution.

Following four consecutive episodes of epistaxis within four days, three of which required nasal tamponade for bleeding control, the patient was referred for hematological evaluation. Psychomotor development was age-appropriate. He had no known allergies or chronic illnesses and was not receiving any chronic or acute medications. He was vaccinated according to the national immunization schedule, without hematoma formation after intramuscular vaccinations. His diet was balanced and age-appropriate, and he attended kindergarten.

**Figure 1:**
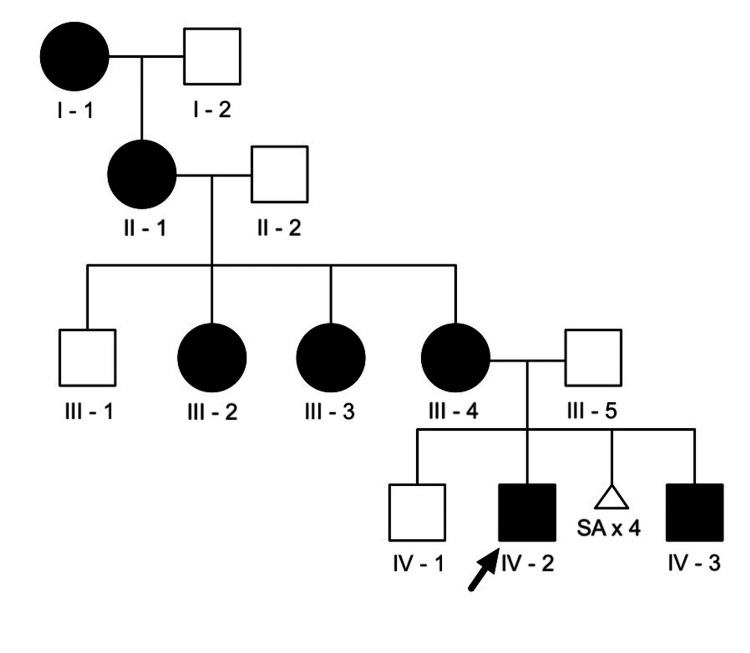
Pedigree chart demonstrating autosomal dominant inheritance The arrow indicates the proband. Roman numerals (I, II, III) indicate generations, whereas Arabic numerals (e.g., 1, 2, 3) identify individual family members within each generation. SA: spontaneous abortion

The patient is of Caucasian ethnicity, and the parents are not consanguineous. Family history revealed multiple female relatives on the maternal side with documented thrombocytopenia and increased bleeding tendency, including heavy menstrual bleeding (HMB). As illustrated in Figure 1, affected family members included the patient’s mother (III-4), her two sisters (III-2 and III-3), the maternal grandmother (II-1), and the maternal great-grandmother (I-1). The patient’s mother had platelet counts ranging from 70 to 120 × 10^9^/L and received platelet transfusion therapy during her first pregnancy after platelet counts decreased below 40 × 10^9^/L. In addition to two full-term pregnancies, she experienced four spontaneous miscarriages during early pregnancy. During adolescence, pseudothrombocytopenia was suspected. During her first pregnancy, IgM antiplatelet antibodies were detected; however, hematological follow-up was irregular, and further diagnostic evaluation was not pursued.

One maternal aunt had recurrent spontaneous miscarriages in early pregnancy, while the other experienced cutaneous hemorrhages. Both the maternal grandmother and great-grandmother reported HMB. The patient’s older brother had intellectual and speech development difficulties. The exact cause of the brother’s intellectual and speech development difficulties remained unknown, and he had no documented thrombocytopenia. In contrast, the younger brother developed thrombocytopenia after birth that resolved spontaneously; however, no subsequent hematological evaluation was performed.

Physical and neurological examinations were unremarkable except for several hematomas in different stages of resolution located on the shins. No pallor, lymphadenopathy, hepatosplenomegaly, or dysmorphic features were observed, and the child had normal growth and development for age. Initial laboratory evaluation revealed a platelet count of 124 × 10^9^/L (reference range: 150-400 × 10^9^/L) and a mean platelet volume (MPV) of 14.7 fL (reference range: 7.0-10.4 fL), while hemoglobin (121 g/L; reference range: 109-138 g/L), total leukocyte count (7.8 × 10^9^/L; reference range: 5.0-13.0 × 10^9^/L), and differential leukocyte count (neutrophils 27.3%, lymphocytes 52.1%, monocytes 15%, eosinophils 4.6%, basophils 1.0%) were within normal limits. There was no previously documented history of thrombocytopenia. Peripheral blood smear analysis demonstrated enlarged platelets with platelet anisocytosis.

The patient’s medical history and clinical presentation were not suggestive of ITP, and antiplatelet antibody testing was negative. A comprehensive diagnostic evaluation for secondary causes of thrombocytopenia, including assessment of vitamin levels (vitamin B12, folate, and vitamin D), autoimmune markers (antinuclear antibodies (ANA), anti-double-stranded DNA antibodies, extractable nuclear antigen (ENA) panel, lupus anticoagulant, anticardiolipin antibodies IgG and IgM, anti-β2 glycoprotein I antibodies, anti-thyroid peroxidase antibodies, and anti-thyroglobulin antibodies), and infectious screening, yielded negative results.

Global coagulation studies were within normal limits, and thromboelastography (TEG) demonstrated a normal coagulation profile. Von Willebrand disease type 2B was excluded. Flow cytometric analysis demonstrated normal expression of CD41, CD42b, and CD61 (>70%), making Glanzmann thrombasthenia and BSS-like defects less likely [1]. Platelet function analysis (PFA-100) demonstrated mildly prolonged closure time with epinephrine as the agonist (175 seconds; reference range: 80-150 seconds). Alternative causes of epistaxis, including nasal trauma and mucosal dryness, were excluded.

Given the strongly positive family history, NGS was performed approximately six months after the initial presentation as part of the diagnostic evaluation. Genetic analysis identified a heterozygous likely pathogenic variant in the ACTN1 gene, confirming the diagnosis of ACTN1-related thrombocytopenia, an autosomal dominant inherited macrothrombocytopenia.

During follow-up, recurrent episodes of epistaxis resulted in iron-deficiency anemia secondary to chronic blood loss, for which ferric hydroxide polymaltose complex was initiated at a dose of 3 mg/kg/day. Over the subsequent two-year follow-up period, the frequency of epistaxis and bruising decreased, platelet counts ranged from 111 × 10^9^/L to repeatedly normal values, and no further anemia occurred. The patient was advised to avoid medications affecting platelet function, including nonsteroidal anti-inflammatory drugs, as well as contact sports. Hematology consultation prior to invasive procedures was recommended. Genetic counseling was offered, and segregation analysis of the parents was recommended but not pursued. Regular follow-up at the hematology outpatient clinic was advised. At the time of writing, the patient continues to develop normally without significant bleeding complications.

## Discussion

**Figure 2:**
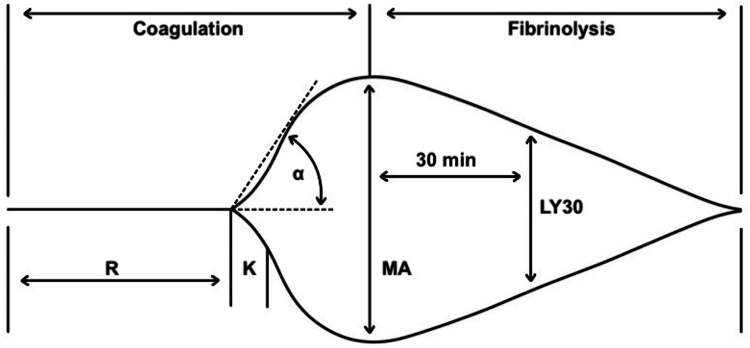
Thromboelastography (TEG) parameters. Schematic representation of R time, K time, α-angle, MA, and LY30. R: reaction time; K: clot formation time; α-angle: rate of clot formation; MA: maximum amplitude; LY30: lysis after 30 minutes Source: Modified from “Thromboelastography parameters” available at Wikimedia Commons (https://commons.wikimedia.org/wiki/File:Thromboelastography_parameters.png), licensed under CC BY.

Classification of bleeding disorders can be challenging because many conditions share similar clinical presentations and laboratory findings [4]. This case highlights how routine laboratory values may be misleading, as platelet counts in hereditary thrombocytopenias can remain within reference ranges or be only mildly decreased. Standard hematologic and coagulation studies are often nonspecific, which may result in delayed diagnosis or inappropriate treatment [5]. In such cases, TEG can provide valuable diagnostic insight by offering a real-time assessment of global hemostatic function and clot viscoelastic properties. TEG evaluates multiple stages of coagulation, including clot initiation, formation, strength, and fibrinolysis, helping differentiate between platelet dysfunction, coagulation factor deficiencies, and hyperfibrinolysis. The principal TEG parameters include the R time, K time, α-angle, maximum amplitude (MA), and LY30, each reflecting a distinct phase of clot formation and stability (Figure 2) [6].

Macrothrombocytopenia has been associated with prolonged K time and reduced α-angle and MA values on TEG (Table 1) [7]. In contrast, all TEG parameters in our patient remained within established reference intervals (R time 5.3 min, K time 1.8 min, α-angle 71°, MA 58 mm, and LY30 6%), consistent with preserved global hemostatic function. One possible explanation is preserved functional activity of macroplatelets despite reduced platelet numbers [8]. Additionally, compensatory effects from other hemostatic factors, including fibrinogen concentration, hematocrit, and plasma coagulation components, may contribute to relatively normal global clot formation [9]. Pre-analytical and technical factors related to TEG testing may also influence the results.

**Table 1:** Interpretation of TEG parameters. TEG: thromboelastography; R: reaction time; K: clot formation time; MA: maximum amplitude; LY30: lysis after 30 minutes; FFP: fresh frozen plasma Source: Modified from reference [6].

Parameter	Reference values	Measures	Interpretation	Treatment
R	4.6-9.1 min	Time until initial fibrin formation, clotting factors	Prolonged: coagulation factor deficiency, anticoagulant therapy	FFP
K	0.8-2.1 min	Time to achieve a certain clot strength, fibrinogen	Prolonged: fibrinogen deficiency, platelet dysfunction	depending on the cause
α-angle	63-78°	Rate of clot formation, fibrinogen	Decreased: hypofibrinogenemia, platelet dysfunction	cryoprecipitate
MA	52-69 mm	Strength of fibrin clot, interaction of platelets (80%) and fibrin (20%) via GPIIb/IIIa	Decreased: thrombocytopenia, platelet dysfunction	platelets
LY30	0-8%	Amplitude decreases due to clot lysis 30 minutes after MA, fibrinolysis	Increased: hyperfibrinolysis	tranexamic acid

Although thrombocytopenia is defined as a platelet count below 150 × 10^9^/L, serial platelet measurements are important in patients with suspected disorders of primary hemostasis. Evaluation should also include assessment for associated platelet function disorders (thrombocytopathies). In children with thrombocytopenia, MPV is an important parameter to consider (Table 2), as it may serve as an initial screening tool for differentiating hereditary thrombocytopenias from acquired causes such as ITP [10]. When interpreted alongside the clinical history, MPV may also help narrow the differential diagnosis among hereditary thrombocytopenias. Hereditary macrothrombocytopenias, including ACTN1-related thrombocytopenia, BSS, and MYH9-related disorders, are typically associated with markedly increased MPV due to the presence of enlarged platelets [11]. In contrast, MPV in ITP is generally normal or moderately increased as a result of increased peripheral platelet turnover and release of younger platelets [10].

**Table 2:** Classification of hereditary thrombocytopenias according to mean platelet volume (MPV) The asterisks (*) and (**) denote condition-specific clinical features within the same table row. WAS: Wiskott‑Aldrich syndrome; BSS: Bernard‑Soulier syndrome Source: Reference ranges and disease associations are adapted from references [12,13].

MPV category	MPV (fL)	Typical conditions	Key clinical features
Low MPV (microthrombocytopenia)	<7 fL	WAS	Immunodeficiency; eczema
Normal MPV	7-10.5 fL	RUNX1- related thrombocytopenia, ANKRD26- related thrombocytopenia, FLI1‑ related thrombocytopenia	Detailed family and bleeding history; predisposition to hematologic malignancies
High MPV (macrothrombocytopenia)	>10.5 fL	BSS, ACTN1‑related macrothrombocytopenia*; MYH9‑related disorders**	*lifelong bleeding tendency; **sensorineural hearing loss, nephropathy, early-onset cataracts

This case highlights the importance of obtaining a detailed personal and family history when evaluating suspected disorders of primary hemostasis. Although the patient’s platelet counts were only intermittently decreased, the bleeding history and positive family history warranted further diagnostic evaluation. Hereditary thrombocytopenias may present with only mild thrombocytopenia or even platelet counts within reference ranges [14]. Therefore, when clinical suspicion for an inherited bleeding disorder is high, early genetic testing should be considered, as it is increasingly accessible and often essential for establishing a definitive diagnosis.

NGS, including whole-exome sequencing (WES), represents a key diagnostic tool in hereditary macrothrombocytopenias. Because the phenotypes of many inherited thrombocytopenias overlap considerably, identification of the underlying molecular defect can improve prognostic assessment and help exclude syndromic conditions such as MYH9-related disorders or Wiskott-Aldrich syndrome [15]. These disorders may be associated with extrahematopoietic manifestations requiring additional surveillance and management. In contrast, ACTN1-related thrombocytopenia is typically associated with mild bleeding symptoms and absence of systemic involvement, emphasizing the importance of precise molecular diagnosis [16].

In this case, the patient carried a heterozygous, likely pathogenic ACTN1 variant inherited in an autosomal dominant pattern. This finding is clinically relevant because several female relatives demonstrated thrombocytopenia and similar bleeding tendencies, suggesting vertical transmission within the family [17]. The ACTN1 gene encodes α-actinin-1, a cytoskeletal protein involved in actin filament organization in megakaryocytes and platelets [8]. Pathogenic variants disrupt normal platelet formation and lead to macrothrombocytopenia with variable bleeding severity, even among affected members of the same family. Approximately 50 pathogenic ACTN1 missense variants have been described to date [18].

The ACTN1 variant identified in our patient, p.Arg320Gln (c.959G>A), is associated with autosomal dominant hereditary macrothrombocytopenia characterized by mild thrombocytopenia, enlarged platelets, and generally low bleeding risk. Previously reported cases suggest a stable hematologic phenotype without significant syndromic manifestations or severe hematologic complications [14,19]. The patient’s mother experienced worsening thrombocytopenia late in pregnancy, which is unlikely to be explained solely by ACTN1-related thrombocytopenia; pregnancy-related conditions such as gestational thrombocytopenia or preeclampsia were considered more likely contributors. However, maternal genetic testing was declined, preventing confirmation of carrier status.

We also included information regarding the neurological difficulties of the patient’s older brother and the history of spontaneous abortions in the patient’s mother and aunt. Based on currently available literature, these findings have not been associated with ACTN1 variants. Although platelets are increasingly recognized to participate in thromboinflammatory pathways through interactions with activated endothelium, leukocytes, and coagulation proteins [20], any potential relationship between ACTN1-related thrombocytopenia and these clinical findings remains speculative. Segregation analysis among family members and genetic counseling were therefore recommended.

Therapeutic options for inherited thrombocytopenias remain largely supportive despite advances in molecular diagnosis [4]. Currently, no curative or gene-specific therapies are available for most inherited bleeding disorders. Management is usually based on preventive measures, including avoidance of antiplatelet medications, maintenance of good dental hygiene, limitation of high-risk activities, and regular hematologic follow-up with monitoring for complications such as iron deficiency. Platelet transfusions, antifibrinolytic agents, or desmopressin are generally reserved for patients with clinically significant bleeding or high-risk procedures [15].

## Conclusions

This case highlights the importance of genetic testing in the diagnosis of inherited bleeding disorders, as routine laboratory evaluation and clinical presentation may remain nonspecific despite a strongly suggestive personal and family history. Although treatment options are largely preventive and supportive, early molecular diagnosis can improve individualized patient management, facilitate appropriate long-term monitoring, and enable timely genetic counseling for affected families. As genetic sequencing technologies become increasingly accessible, early genetic evaluation should be considered in patients with suspected congenital thrombocytopenias, particularly when conventional diagnostic findings are inconclusive.
